# Long-term health- and cost evaluation of two work-oriented rehabilitation models for women on long-term work disability due to common mental disorders or chronic pain – a randomized controlled trial

**DOI:** 10.1186/s12889-026-27634-4

**Published:** 2026-05-06

**Authors:** Anna Finnes, Magnus Johansson, Magnus Helgesson, Åsa Andersén, Erik Berglund, Ingrid Anderzén

**Affiliations:** 1 Department of Public Health and Caring Sciences, Uppsala University, Uppsala, SE- 751 22 Sweden; 2 Division of Insurance Medicine, Department of Clinical Neuroscience, Karolinska Institutet, Stockholm, SE-171 77 Sweden; 3 Academic Primary Health Care Center, Region Stockholm, Stockholm, SE-113 65 Sweden; 4 Centre for Psychiatry Research, Department of Clinical Neuroscience, Karolinska Institutet, Stockholm, SE-113 64 Sweden

**Keywords:** Vocational rehabilitation, Acceptance and commitment therapy, Cost-effectiveness analysis, Absenteeism, Women, Ordered beta regression

## Abstract

**Background:**

Long-term outcomes of work rehabilitation for individuals on extended sick leave due to common mental disorders or chronic musculoskeletal pain remain insufficiently studied. This randomized controlled trial evaluated the long-term effects on work disability and cost-effectiveness over eight years, and health outcomes over ten years, comparing (1) unimodal Acceptance and Commitment Therapy (ACT) and (2) multidisciplinary assessment and treatment program including ACT (TEAM) with standard care (Control).

**Methods:**

Work disability days were analysed using ordered beta regression, and health outcomes were assessed using mixed models for repeated measures. Results are presented as estimated marginal means.

**Results:**

Both intervention groups demonstrated lower model-predicted median work disability days at all time points compared to Control, but the differences were only statistically significant for the TEAM group at years four, seven, and eight. Both ACT and TEAM interventions showed superior effects on psychiatric symptom reduction relative to Control at one- and two-years follow-up.

**Conclusion:**

The findings highlight the importance of extended follow-up to fully capture the effects of rehabilitation efforts. The results support the consideration of both rehabilitation models for women with prolonged work disability, with the choice between them potentially guided by available resources, individual patient complexity, and a stepwise approach to care.

**Trial registration:**

The study was retrospectively registered at the Clinicaltrials.gov Register Platform on November 15, 2017 (ID NCT03343457).

**Supplementary Information:**

The online version contains supplementary material available at 10.1186/s12889-026-27634-4.

## Background

Common mental disorders, including depression, anxiety, and adjustment disorders, as well as chronic musculoskeletal pain, represent substantial public health challenges globally. These conditions place a significant burden on welfare systems and have considerable economic implications for both individuals and society [1–3]. They are also strongly associated with long-term work disability (WD) and are frequently complicated by co- or multimorbidity, which adversely affects quality of life, functional capacity, and prognosis [4]. The resulting complexity in healthcare and service utilization is a major contributor to both direct and indirect healthcare costs, which tend to increase over time [5].

In response to rising costs and prolonged sickness absence, Sweden implemented substantial reforms to its social insurance policy in 2008. These reforms introduced an upper time limit for sickness benefits, mandated assessments of work ability, and established fixed time points for initiating rehabilitation efforts [6]. Under the new policy, individuals who reached the time limit lost eligibility for sickness benefits for a period of three months and were required to undergo a work ability assessment conducted by the Swedish Public Employment Service (SPES) in collaboration with the Swedish Social Insurance Agency (SSIA).

Multi-domain interventions encompassing healthcare provision, service coordination (e.g., workplace involvement), improved communication between stakeholders, development of return-to-work (RTW) plans, and work accommodation, have been recommended to reduce time to RTW for individuals with pain-related and mental health conditions [7]. However, for patients with multimorbidity, evidence of positive effects on clinical outcomes, healthcare utilization, medication adherence, patient behaviours, provider behaviours, or cost-effectiveness remains limited [8]. More recently, transdiagnostic approaches targeting shared underlying processes have been proposed to improve outcomes in individuals with concurrent pain and emotional disturbances [9]. One such approach is Acceptance and Commitment Therapy (ACT), an empirically supported form of cognitive behavioural therapy (CBT) that targets transdiagnostic processes such as psychological inflexibility and experiential avoidance [10]. ACT aims to enhance psychological flexibility, defined as the ability to persist in or change behaviour in accordance with personally valued goals, depending on the context. ACT has demonstrated efficacy in reducing symptoms of both mental disorders and chronic pain [11], and has shown positive effects on RTW outcomes for up to two years [12], as well as cost-effectiveness in transdiagnostic vocational rehabilitation for individuals on long-term sick leave [13]. The use of a transdiagnostic model such as ACT offers several advantages: it enables treatment of individuals with diverse medical conditions, allows clinicians to specialize in a single therapeutic framework, and can be implemented in both unimodal and multidisciplinary settings [14].

We have previously reported findings from a randomized controlled trial involving women whose sickness benefits were discontinued due to the aforementioned policy changes [15–19]. The trial compared (1) unimodal psychological treatment (ACT) and (2), multidisciplinary assessment and treatment including ACT (TEAM) with standard care (Control), within the context of the mandated work ability assessment. Results indicated that both rehabilitation models improved employability at the one-year follow-up [16] and were more effective than Control in reducing health-related symptoms [17]. However, the long-term effects of these interventions remain to be evaluated regarding work disability and effects on psychiatric symptoms.

The primary aim of the present study was to examine the long-term effects of the ACT and TEAM interventions on WD up to eight years and health outcomes at two and ten years after baseline. A secondary aim was to conduct a limited cost-effectiveness analysis comparing the ACT and TEAM intervention groups to the Control group at one-, two-, and eight-years follow-up.

## Methods

### Trial design and participants

A randomized controlled trial with three arms, ACT, TEAM, and Control, was designed following CONSORT guidelines.

**Fig. 1:**
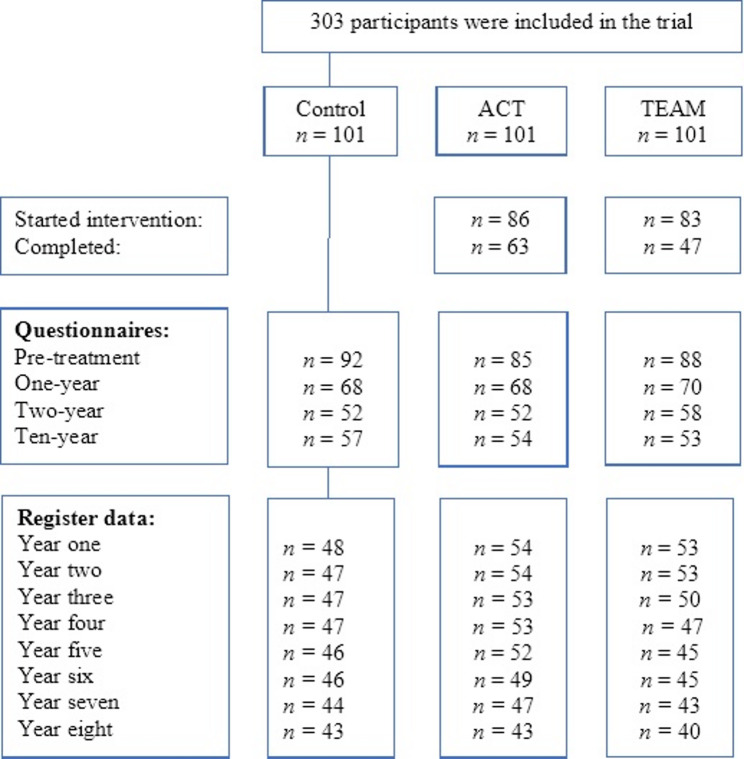
Trial flow diagram

The participant flow is illustrated in Fig. 1. The targeted population was women in Uppsala County subjected to workability assessments due to policy changes from 2010 to 2011. Medical records from the SSIA were reviewed, and those meeting the inclusion criteria were invited to participate in the study. The inclusion criteria were female sex, being of working age (20–64 years), on long-term sick leave due to musculoskeletal pain and/or common mental disorders and approaching the maximum time limit for sickness benefits. Exclusion criteria included severe mental health issues, current involvement in psychotherapy, or participation in another structured vocational rehabilitation program. Those who consented to participate were randomized by an administrator in blocks of three into one of the treatment arms. The inclusion procedure has been described in greater detail elsewhere in a previous publication [18].

As noted in the one-year follow-up publication [17], a procedural error led to participants being informed of their group allocation prior to baseline assessment, potentially influencing baseline symptom ratings, particularly in the control group. Therefore, this paper aims to adapt the statistical analysis approach to account for potential baseline imbalances between groups as well as long term attrition.

### Interventions

Participants were randomized to one of three intervention arms: unimodal ACT treatment including clinical assessment and treatment by a psychologist (ACT), individual assessment and treatment provided by a multidisciplinary team consisting of a psychologist, physician, occupational therapist, and social worker (TEAM), and Control which consisted of standard care. All groups participated in the collaborative approach offered by the SPES and SSIA which aimed to assess work ability Control. The interventions have been detailed elsewhere [17].

### Outcome measures

*The primary outcome measure* was WD quantified as SA days with sickness benefits and days on DP. Both SA and DP can be granted on a full-time (100%) or part-time basis (25%, 50%, 75%) of ordinary working hours, i.e., it is possible to have both part-time SA and DP benefits simultaneously. SA/DP was calculated as overall WD net days, e.g., whole-day equivalents of the sum of part-time and full-time absences. Data was collected through Statistics Sweden registers.

*Secondary outcome measures* included health measures and psychological processes. General health was measured with the General Health Questionnaire [20]. Anxiety and depression levels were measured with the two subscales in the Hospital Anxiety and Depression Scale [21, 22]. Pain level was estimated by a single question from the Örebro musculoskeletal pain questionnaire: “Do you have pain?” [23]. In case of a positive answer, the respondent was asked to grade pain intensity on a scale ranging from 0 = no pain to 10 = unbearable pain. Satisfaction with life was measured using the Satisfaction with Life scale [24]. Finally, self-efficacy was measured with the General Self-Efficacy Scale [25]. Internal consistency (Cronbach’s α) for the current sample was > 0.7 for all scales at two- and ten-year follow-ups (See Supplementary Table 1).

### Statistical and data analysis

Analyses were carried out in R version 4.5.3 [26]. Due to WD data having known lower and upper bounds (ranging from zero to n days within a time frame) with disproportionate counts at minimum and maximum values during a time period (zero- and one-inflation), ordered beta regression was used [27] with the R package `glmmTMB` [28]. Model residuals were evaluated using the R package `DHARMa` [29]. Results are reported as group contrasts with estimated marginal means (EMM), produced with `marginaleffects` [30]. EMM are marginal means of model predicted values based on a reference grid with continuous covariates held at their means and all combinations of categorical variables. Cumulative counts of WD days were analyzed cross-sectionally at each year until eight years after baseline, with each year adding to the previous. Covariates were age (grand mean centered), employment status, and diagnosis at study enrolment. Confidence intervals were based on 5000 non-parametric bootstrap iterations.

Self-rated secondary outcomes were evaluated using ordinal sum scores from rating scales treated as interval-level data. Each outcome was modeled using a separate mixed model for repeated measures [31] with an unstructured covariance matrix for time within subjects, which was allowed to vary across treatment groups [32]. The MMRMs were conducted using the R package `*mmrm`* [33], utilizing restricted maximum likelihood and the Kenward-Roger method to adjust the degrees of freedom and coefficients’ covariance matrix [34]. All MMRM models employed the same formula with the grand mean centered outcome pre-measurement as a covariate interacting with the categorical time variable [35, 36]. The treatment group was specified as having an interaction with time. Covariates were absence from work in years, sick-leave status, diagnosis, employment status, and grand mean centered age. Results averaged over the levels of covariates are reported as EMM for each group at each follow-up measurement, alongside EMM contrasts between groups, using the R package `*emmeans`* [37]. Figures were created using `ggplot2` [38] and include 84% CIs to simplify visual interpretation of statistical significance in group comparisons as non-overlapping CIs [39].

See Supplementary materials for details on model specifications and a more extensive presentation of results. Both primary and secondary statistical models used the recommendations from the United States Food and Drug Administration for including covariates when analyzing data from randomized controlled trials [40], applying the ANHECOVA (analysis of heterogeneous covariance) method [41–43]. As noted earlier, differences between treatment groups and attrition also warranted covariate control where the treatment was interacted with relevant covariates. Thus, no unadjusted results are reported. As a sensitivity analysis for secondary outcomes, we also used a simpler setup with cross-sectional (yearly) models using overlap weights to balance the treatment groups. This is described and reported in Supplementary Materials, where covariate balance plots also are available in Supplementary Fig. 1–3. These analyses made use of R packages `cobalt` [44] and `WeightIt` [45].

A limited within-trial cost-effectiveness analysis was performed. *Operating cost estimates* were derived from the average cost for time spent by each professional including assessments, treatment, administration and collaboration with other stakeholders. Costs were based on the cost per visit to health care providers in Region Uppsala from the Cost per Patient Database [46]. For participants in TEAM, time for professionals participating in team conferences, including feedback to participants, was also included. A total intervention cost was calculated for each participant. *Effect estimates* were estimated WD days. *The cost-effectiveness analysis* was undertaken from an intervention cost perspective, where the cost for the control group was set to zero, assuming that healthcare consumption outside the project was equal between the three groups. The incremental cost-effectiveness ratio (ICER) was calculated for each group comparison using the general formula (∆^C1^-∆^C2^) / (∆^E1^-∆^E2^), reflecting the difference in average cost per reduced WD day between the control group and each intervention group. Non-parametric bootstrap was conducted using 1000 iterations. In each bootstrap iteration, the effect was estimated using ordered beta regression and the cost using linear regression, both using covariates as previously described. The estimated marginal mean effect and cost were then used to calculate the ICER. Results were summarized in cost-effectiveness planes and the probability of each intervention being cost-effective compared to control was reported based on an assumed willingness-to-pay (WTP) set at 100 EUR per reduced WD day.

## Results

When we asked for consent to participate in the 10-year follow-up and to extract register data, five of the original 308 participants withdrew their consent (Control=three, ACT = one, TEAM = one), leaving 303 participants at baseline for this analysis. A total of 164 (Control = 57, ACT = 54, TEAM = 53) consented to participate in the long-term follow-up. For the withdrawal of registry data, 155 participants consented by returning the completed survey in 2020 and were included in the analyses: 48 in the control group, 54 in the ACT intervention, and 53 in the TEAM intervention (see Fig. 1). Demographic characteristics are presented in Table 1.

**Table 1:** Demographic characteristics of all consenting participants at baseline and for the subsample that consented to participate in the long-term follow-up and provided answers to at least one survey outcome

Characteristic	Total		Control		ACT		Team	
	Baseline	Follow-up	Baseline	Follow-up	Baseline	Follow-up	Baseline	Follow-up
	*N* = 303^1^	*N* = 154^1^	*N* = 101^1^	*N* = 44^1^	*N* = 101^1^	*N* = 50^1^	*N* = 101^1^	*N* = 50^1^
Age Median [IQR]	48 [42, 55]	51 [44, 56]	48 [42, 54]	50 [43, 54]	47 [42, 54]	50 [43, 57]	51 [44, 56]	53 [46, 57]
Birth country n (%)								
Sweden	211 (80)	122 (86)	67 (75)	39 (87)	70 (81)	40 (83)	74 (83)	43 (88)
Other	53 (20)	20 (14)	22 (25)	6 (13)	16 (19)	8 (17)	15 (17)	6 (12)
Education n (%)								
Primary school	44 (20)	19 (15)	16 (21)	7 (18)	11 (15)	3 (6.8)	17 (23)	9 (20)
Secondary school	97 (44)	59 (46)	32 (43)	14 (36)	32 (43)	19 (43)	33 (45)	22 (50)
University	81 (37)	49 (39)	27 (36)	18 (46)	31 (42)	22 (50)	23 (32)	13 (30)
Civil state n (%)								
Married	181 (69)	103 (73)	60 (68)	33 (75)	59 (69)	33 (69)	62 (70)	37 (76)
Single	82 (31)	38 (27)	28 (312)	11 (25)	27 (31)	15 (31)	27 (30)	12 (24)
Employment status n (%)								
Employed	195 (64)	107 (69)	66 (65)	29 (66)	59 (58)	34 (68)	70 (69)	38 (76)
Unemployed	108 (36)	47 (31)	35 (35)	15 (34)	42 (42)	16 (32)	31 (31)	12 (24)
Diagnosis n (%)								
Pain	111 (37)	62 (40)	40 (40)	17 (39)	33 (33)	19 (38)	38 (38)	25 (50)
Psych	100 (33)	51 (33)	28 (28)	14 (32)	42 (42)	20 (40)	30 (30)	14 (28)
Pain and psych	92 (30)	41 (27)	33 (33)	13 (30)	26 (26)	11 (22)	33 (33)	11 (22)
Sickleave status n (%)								
Fulltime	153 (51)	63 (41)	52 (51)	18 (41)	54 (53)	23 (46)	47 (47)	17 (35)
Parttime	149 (49)	90 (59)	49 (49)	26 (59)	47 (47)	27 (54)	53 (53)	32 (65)
Unknown	1	1	0	0	0	0	1	1
Years on sickleave Median [IQR]	7.82 [5.09, 9.91]	7.88 [5.09, 10.06]	8.05[4.75, 10.13]	8.98[6.31, 10.21]	7.86 [5.57, 9.59]	6.82 [4.58, 9.66]	7.42 [5.11, 9.71]	8.19 [5.09, 10.06]

^1^n (%); Median = Median, IQR [Q1, Q3]. Background variables had missing data, see Fig. 1

The original study sample had a mean duration of work disability of approximately 7.8 years prior to enrolment. At baseline, approximately half of the participants received full (100%) benefits, more than one-third were not affiliated with an employer, around one in five had a low level of education, and approximately one-third presented with both a psychiatric disorder and chronic pain (see Table 1). The subsample participating in the long-term follow-up was similar to the original cohort with respect to key baseline characteristics. Distributions of benefit status, employment affiliation, education, and health characteristics were comparable between the total sample and the follow-up subsample (Table 1). However, there were baseline differences between groups in demographic characteristics and pre-treatment outcome variables, which became more pronounced at follow-up (see Supplementary materials, Figs. 1, 2 and 3).

**Fig. 2:**
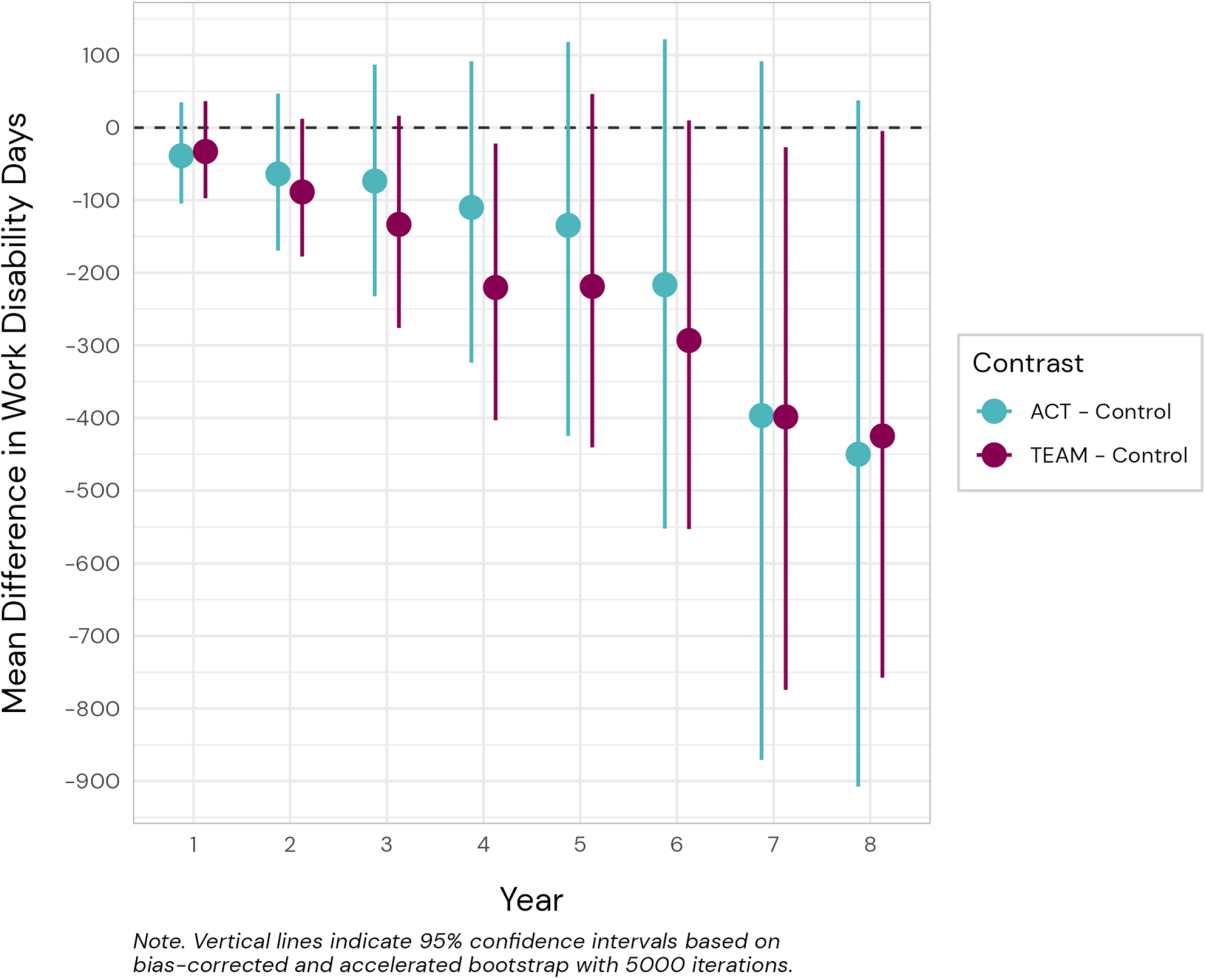
Visualization of group contrasts of treatment vs. control based on estimated marginal means and 95% confidence intervals for work disability days across eight years follow-up

**Fig. 3:**
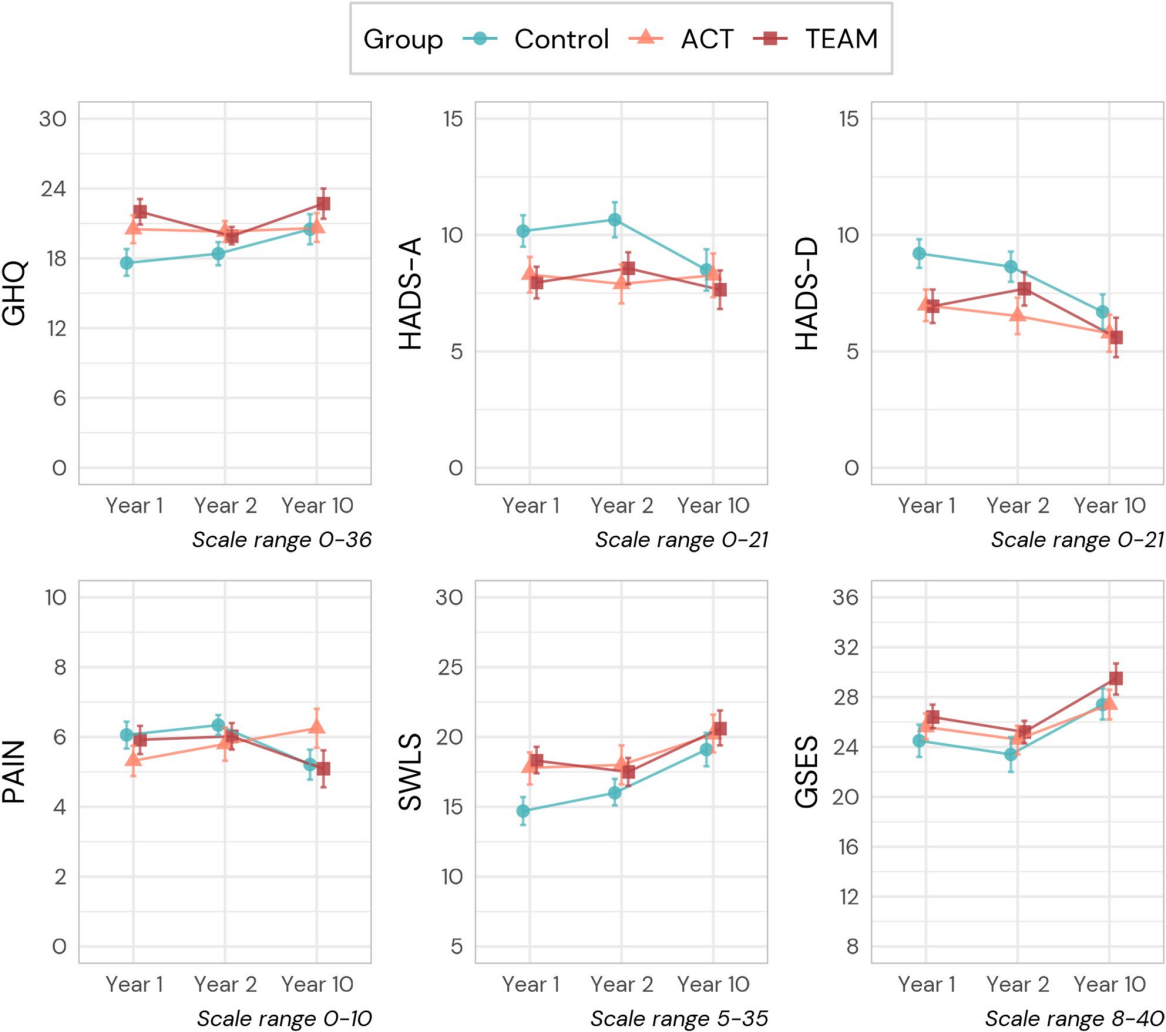
Visualization of estimated marginal means and 84% confidence intervals for secondary outcome measures at one, two-, and ten-year follow-up. Note: HADS-D = Hospital Anxiety Depression Scale - Depression; HADS-A = Hospital Anxiety Depression Scale - Anxiety; SWLS = Satisfaction With Life Scale; GHQ = General Health Questionnaire; GSE = General Self-Efficacy Scale; PAIN = Örebro Musculoskeletal Pain Scale

All participants who reached retirement age (65) during the follow-up period were excluded. Consequently, the analyses involved slightly fewer participants over the years. A drop-out analysis is presented in Supplementary materials.

### Outcomes

#### Primary outcome

Results are summarized in Fig. 2 and Supplementary Table 2. Based on estimated marginal means, both TEAM and ACT intervention groups had lower model predicted mean counts of WD days at each cumulative time point compared to control. The results only showed statistically significant differences from control for TEAM at years four, seven, and eight. Bootstrap estimates of comparatively fewer cumulative WD days were − 220 at year four (95% CI [-403, -22]), -399 days at year seven (95% CI [-774, -27], and 425 days at year eight (95% CI [-758, -5]), respectively. The R^2^ statistics for yearly models ranged from 0.05 to 0.12, with a mean of 0.08.

#### Secondary outcomes

The results of the analyses are presented in Fig. 3 and Supplementary Table 3. Observed means and standard deviations are presented in Supplementary Table 3. For self-rated general health, the EMM difference on the GHQ scale after one year was 4.37 units higher in TEAM compared with Control (*p* < 0.001), while ACT was 2.87 units higher (*p* = 0.032). There were no differences after two or ten years. Self-rated satisfaction with life measured with the SWLS showed a similar pattern to that of GHQ. For Anxiety, there were large differences in HADS Anxiety subscale ratings vs. Control for both ACT (EMM = -1.88, *p* = 0.019) and TEAM (EMM = -2.21, *p* < 0.003) at one year, which were maintained at two years (ACT: EMM = -2.76, *p* = 0.001; TEAM: EMM = -2.08, *p* = 0.009) but not after ten years. A similar trend was observed in depression ratings (HADS Depression) with large differences between groups in ratings at year one (ACT: EMM = -2.23, *p* = 0.001; TEAM: EMM = -2.27, *p* = 0.002) but only for ACT after two years (ACT: EMM = -2.12, *p* = 0.008) and no statistically significant differences at ten years follow-up.

There were no clinically meaningful differences in EMM for any of the groups at any time point regarding self-rated pain levels and general self-efficacy. The R^2^ statistics for secondary outcome models ranged from 0.40 to 0.52, with a mean of 0.47.

### Cost-effectiveness

Cost-effectiveness analyses for ACT and TEAM compared with Control, respectively, were conducted for year one, two and eight.

#### ACT

**Fig. 4:**
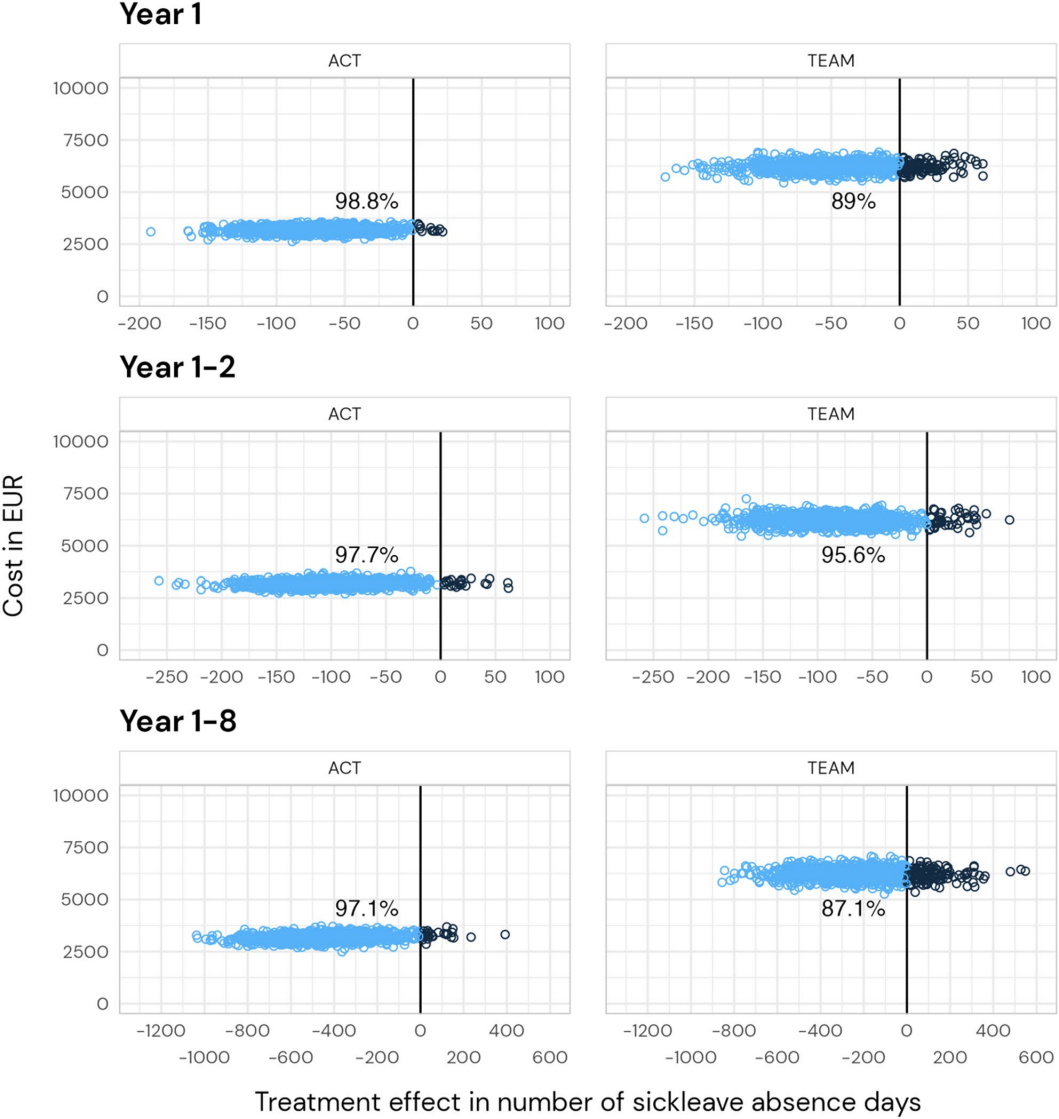
Cost-effectiveness planes for cumulative effects at year one, two, and eight from an intervention costs perspective. Percentages indicate the proportion of bootstrap estimates that have less WD days compared to the control group

One hour of assessment by a psychologist was set to a cost of €190, and two hours of administrative work €380. Collaboration with other authorities was estimated to have an average cost per participant of €236. Home visits by psychologists were estimated to have an average cost of €662. In total, the ACT intervention entailed a median cost of €2 988 per participant (IQR [2 418, 4 128], range [1 468, 5 838]). *Effect*: The estimated number of average WD days was 121 (95% CI [93, 150]) at year one, 253 (95% CI [198, 309]) at year two and 947 (95% CI [708, 1187]) at year eight. *Cost-effectiveness* planes are shown in Fig. 4. When the willingness to pay was 100 Euros per day, the probability of a cost-effective intervention for ACT was 87.5% after one year, 91.8% after two years, and 96.2% after eight years.

#### TEAM

The costs for the initial multidisciplinary assessment (physician (cost/visit €401), psychologist (€190), social worker (€86), and occupational therapist (€187), added up to a total of €864. Additional costs were team conference (€864), feedback to the participant (€189), collaboration with other stakeholders (€297), administration (eight hours per participant, €1 728), and home visits (€604). In total, the median cost for the TEAM intervention was €6 306 per participant (IQR [5 294, 6 868], range [4 546, 10 013]). *Effect*: The estimated number of average WD days was 147 (95% CI [112, 181]) at year one, 266 (95% CI [211, 320]) at year two and 1 144 (95% CI [885, 1402]) at year eight. *Cost-effectiveness* planes are shown in Fig. 4. When the willingness to pay was 100 Euros per day, the probability of a cost-effective intervention for TEAM was 34.1% after one year, 67.5% after two years, and 79.8% after eight years.

## Discussion

The findings of this study build on the previously reported effects of multi-domain rehabilitation models. One model focused on psychological treatment (ACT), where a psychologist assessed and delivered treatment, while the other was multidisciplinary, utilizing different professionals (TEAM) for assessment and treatment delivery. The study population was characterized by a long history of work disability and multiple barriers to return to work, including a high proportion of participants receiving full benefits, lacking employer affiliation, and presenting with both psychiatric and pain-related conditions. The long-term follow-up sample was broadly comparable to the original cohort, suggesting that the findings are likely representative of this population, although selection effects due to attrition cannot be ruled out. Regarding the primary outcome WD days, there were favorable long-term effects associated with the TEAM intervention compared to Control. The estimates indicate a continuous decrease in cumulative WD days for both interventions compared to Control, with statistically significant differences at years four, seven, and eight. Both ACT and TEAM were highly cost-effective concerning the costs of the intervention related to WD days compared to Control. Both interventions showed superior effects on psychiatric symptoms compared to Control for at least two years post-randomization. These effects diminished over time and were no longer significant at the 8-10-year follow-up.

There are few long-term follow-ups of effects after rehabilitation in the literature. This study indicates that there may be positive effects on cumulative WD days up to eight years after rehabilitation is terminated, which is a pattern seen in other studies. For women specifically, a previous study on pain-related multidisciplinary rehabilitation demonstrated positive effects on WD a decade after rehabilitation and with substantial cost-saving effects. However, these effects emerged only after a few years [47]. In another publication from the current trial, long-term effects on work engagement were explored, showing that work engagement increased over time for ACT and TEAM, although the results indicated some time-lag effect of the interventions [19]. Another trial showed that a more extensive multidisciplinary intervention was associated with fewer days of WD than treatment as usual and a lighter form of rehabilitation for women, but not for men, with chronic widespread pain up to 54 months follow-up [48]. Multidisciplinary rehabilitation for pain has also been associated with more days of sickness absence and less work participation at five years follow-up [49]. This highlights the need for more extended follow-up periods to fully capture the effects of vocational rehabilitation efforts.

The interpretation of the present findings is complicated by significant pre-treatment differences between the intervention groups and the control group. Due to a procedural issue, participants were aware of the outcome of randomization when they completed the pre-treatment survey, which may have influenced their responses. These differences were more pronounced for the TEAM group, whose participants reported lower levels of anxiety, better general health, and higher self-efficacy compared to both the Control and ACT groups at baseline. Self-efficacy - reflecting greater confidence in one’s capacity to take on challenging life events - is considered a robust predictor of return to work [50, 52], and may therefore have contributed to the favourable long-term development in WD observed for TEAM.

For mental disorders, long-term follow-up of work-focused cognitive behaviour therapy and individual job support showed that a subgroup of participants most at risk for permanent work exclusion had higher income, higher work participation, and more months without benefits compared to the control group up to 46 months after rehabilitation [51]. In the present study, both interventions demonstrated favourable long-term effects in a population at high risk for permanent work exclusion. A stepwise care model may be warranted, in which a unimodal psychological intervention such as ACT serves as a first-line option, with multidisciplinary rehabilitation reserved for individuals whose needs are not sufficiently addressed by a focused approach. To further understand individual differences, individual benefits from unimodal or multidisciplinary interventions could be mapped out to provide evidence for a stepwise care model for assessing needs and following rehabilitation efforts for individuals on long-term sick leave.

### Strengths and limitations

The use of estimated marginal means improves the generalizability of results, as it reflects the results of balanced demographic groups across conditions. EMM also has the advantage of expressing results on the scale of the outcome, which makes it easy to interpret, especially when the outcome is something as concrete as days. While we were unable to fit a longitudinal model of WD days across time, using ordered beta regression in cross-sectional models is an important methodological improvement compared to how this type of data is usually mistreated in various ways [50]. Since the data has known lower and upper bounds, often with inflation of counts at zero and the upper bound (See Supplementary materials Fig. 1), the statistical model needs to take this into account. A limitation with the current version of the ordered beta regression R package is when data contains mid-inflation, which in this setting could be caused by a relatively large proportion participants on 50% sick leave, this will be underestimated by the statistical model. This phenomenon was also seen in the posterior predictive plots from our models, but to a minor extent.

There are several important limitations to consider when interpreting the findings of this trial. First, as reported in a previous publication [17], a relatively substantial proportion of participants discontinued the interventions before completion, particularly in the TEAM arm. This higher rate of discontinuation may have influenced the effects of the multidisciplinary intervention, although the direction of this influence is uncertain. It is plausible that individuals with more severe or complex problems were over-represented among those who dropped out, in which case their continued participation could have attenuated rather than strengthened the observed effects. Conversely, if dropout was unrelated to severity, the results may underestimate the intervention’s full potential. The impact of differential attrition on the TEAM estimates should therefore be interpreted with caution. Moreover, given the large time span of the follow-up, which is inevitably associated with attrition, covariate adjustment is necessary not only to generate precise estimates but also to produce correct estimates. However, it was not possible to link cost estimate data with register data, which meant that pre-treatment survey measures could not be included as covariates. This constitutes a major limitation, given the observed pre-treatment differences between TEAM and the other groups in levels of anxiety, general health, and self-efficacy. To mitigate the impact of missing data and enhance statistical power, baseline covariates were included in accordance with FDA guidelines for secondary analyses.

The inability to link data also constrained the scope of the cost-effectiveness analysis, which could not incorporate comprehensive healthcare utilization during follow-up. As a result, the cost-effectiveness analysis was conducted from an intervention cost perspective, accounting only for intervention-related costs. A significant limitation is the exclusion of broader societal costs, such as total healthcare consumption, productivity losses, and municipal income support for individuals not eligible for health insurance benefits. To avoid overestimating the cost-effectiveness of the active interventions, the control group was assigned a healthcare cost estimate of zero. The cost-effectiveness results should be interpreted considering the large confidence intervals associated with the effect estimates, particularly at later follow-up time points, which in turn affects the ICER estimates. Importantly, while both interventions were cost-effective relative to Control, the lower intervention costs associated with ACT mean that in scenarios where the marginal benefit of TEAM over ACT is small, as the present findings suggest, the ACT intervention may yield a more favourable cost-effectiveness ratio, a consideration that is particularly relevant for policy decisions regarding resource allocation.

### Generalizability

The population targeted in this study is distinct in that participants had a history of very long sick leave (mean duration: 7.5 years) and were affected by a significant shift in social insurance policy and regulation. Prior to this policy change, the Swedish health insurance permitted extended periods of sickness benefits with limited or no mandatory assessments of work ability during long-term sick leave. The subsequent policy reform introduced fixed time points for reassessing work ability, aiming to reduce long-term dependence on sickness benefits. Although the policy changes likely increased stress in the study population, their impact on the sample may have diminished over follow-up as the new policy became established practice. The observed effects may therefore be attributable to the additional support provided to participants in the intervention groups. This support may have played a role in helping participants cope with the stress associated with recent policy changes and their vulnerable social and economic circumstances. Moreover, the ACT-based intervention, which was offered in both intervention arms, may have contributed by fostering psychological flexibility and supporting participants in improving daily functioning despite ongoing stressors. Altogether, this affects the generalizability of the results to current days. That both interventions performed similarly in many regards, may in part reflect the shared ACT component, which could represent a core mechanism of change common to both models.

Further, few participants had employers who were actively engaged or workplaces with capacity for early adaptations at inclusion. This may partly explain the larger cost-effectiveness effects, as relative cost difference between the interventions and Control is larger further along the trail toward permanent work exclusion. Sickness absence and reduced work functioning while having an engaged employer might be more transient states, with possibly less potential for these types of interventions.

Finally, the study sample was restricted to women, which limits the generalizability of the findings to the broader population. However, given that women are disproportionately represented among individuals with long-term sickness absence, the results remain highly relevant for this subgroup.

## Conclusion

Studies that evaluate long-term, real-life functional outcomes are essential for strengthening the evidence base of rehabilitation approaches. This study contributes to the existing literature by employing a longer follow-up period than most previous research in this area. The duration of treatment effects is a critical factor, as any initial differences between intervention and control groups may diminish over time.

To our knowledge, this is the first study to compare the long-term effects of a unimodal psychological intervention and a multidisciplinary intervention to usual care among women with long-term WD. Both interventions showed favourable long-term trajectories on WD days relative to Control, with TEAM reaching statistical significance and effects persisting up to eight years post-randomization. Both interventions were also cost-effective and showed comparable benefits on psychiatric symptoms. The interpretation of TEAM’s statistical significance in WD days at years four, seven, and eight is tempered by pre-treatment differences in self-efficacy, anxiety, and general health that could not be adjusted for in the WD analysis. Taken together, these findings indicate that the less resource-intensive ACT intervention remains a viable and potentially preferable alternative from a cost-effectiveness standpoint, particularly in settings with limited access to multidisciplinary resources. The results support the consideration of both interventions within rehabilitation practice, with the choice between them potentially guided by available resources, individual patient complexity, and a stepwise approach to care.

## Supplementary Information

Supplementary Material 1.

## Data Availability

The datasets generated and analyzed during the the present study are not publicly available due to confidentiality and ethical considerations. Data availability will require ethical approval from the Swedish Ethical Authority.

## Abbreviations

ACT: Acceptance and Commitment Therapy
CBT: Cognitive behavioural therapy
CI: Confidence interval
CMD: Common mental disorders
CONSORT: Consolidated Standards of Reporting Trials
DP: Disability pension
EMM: Estimated marginal means
FDA: United States Food and Drug Administration
GHQ: General Health Questionnaire
GSES: General Self–Efficacy Scale
HADS: Hospital Anxiety and Depression Scale
ICER: Incremental cost–effectiveness ratio
IQR: Interquartile range
MAR: Missing at random
MMRM: Mixed model for repeated measures
NMAR: Not missing at random
PAIN: Örebro Musculoskeletal Pain Scale
RCT: Randomized controlled trial
RR: Risk ratio
RTW: Return to work
SA: Sickness absence
SE: Standard error
SPES: Swedish Public Employment Service
SSIA: Swedish Social Insurance Agency
SWLS: Satisfaction With Life Scale
TEAM: Multidisciplinary assessment and treatment including Acceptance and Commitment Therapy
WD: Work disability
WTP: Willingness to pay
