# Supplementary material for: Long-term health- and cost evaluation of two work-oriented rehabilitation models for women on long-term work disability due to common mental disorders or chronic pain – a randomized controlled trial
Source: BMC Public Health. 2026 May 6;26:1478. doi: 10.1186/s12889-026-27634-4 (PMC13147580; doi:10.1186/s12889-026-27634-4)
Supplement: Supplementary file 1 — Supplementary Material 1. [file 12889_2026_27634_MOESM1_ESM.docx]

## Supplementary materials

| **Supplementary Table 1.** Internal consistency estimates (Cronbach’s α) for secondary outcome scales at two- and ten-year follow-up. | | |
| --- | --- | --- |
| Scale | Cronbach’s α | |
|  | Two-year follow-up | Ten-year follow-up |
| General Health Questionnaire | .69 | .83 |
| HADS Anxiety Subscale | .87 | .89 |
| HADS Depression Subscale | .90 | .78 |
| Satisfaction with Life scale | .90 | .78 |
| General Self-Efficacy Scale | .96 | .95 |

Figures 1-3 present unadjusted covariate balance with pairwise comparisons. These were made using the R package `cobalt` (Greifer, 2026) and the function `love.plot`. In each subplot, the reference group is the right-hand group in the subplot label. For instance, in ACT vs. Control, a dot on the vertical solid line indicates perfect balance, while the hads_d dot to the left of the dashed vertical line (representing a 0.1 standardized mean difference) indicates that the ACT group had lower ratings on hads_d than control.

**
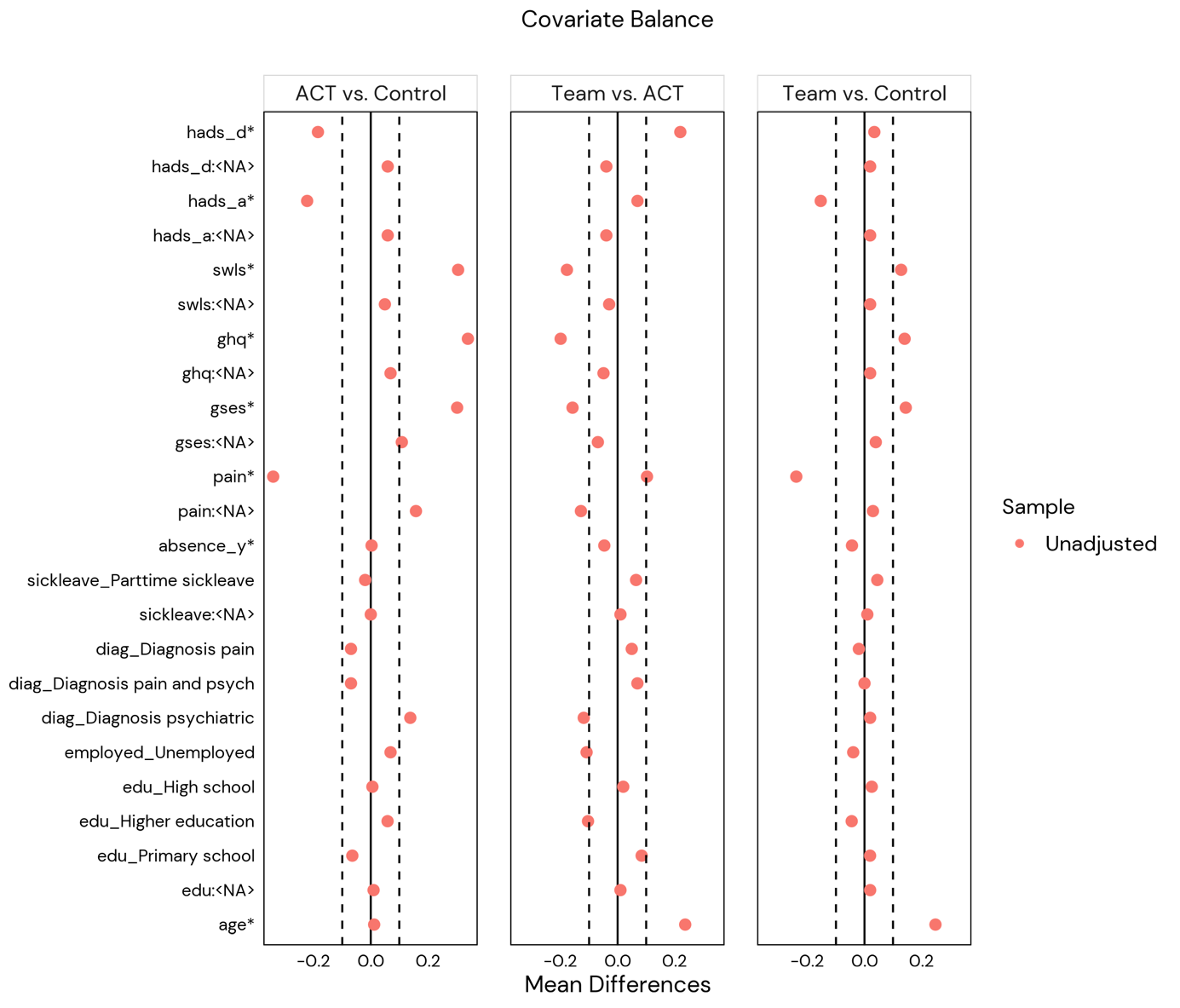
**

**Supplementary Figure 1.** Covariate balance plot at pre-treatment for secondary outcomes.


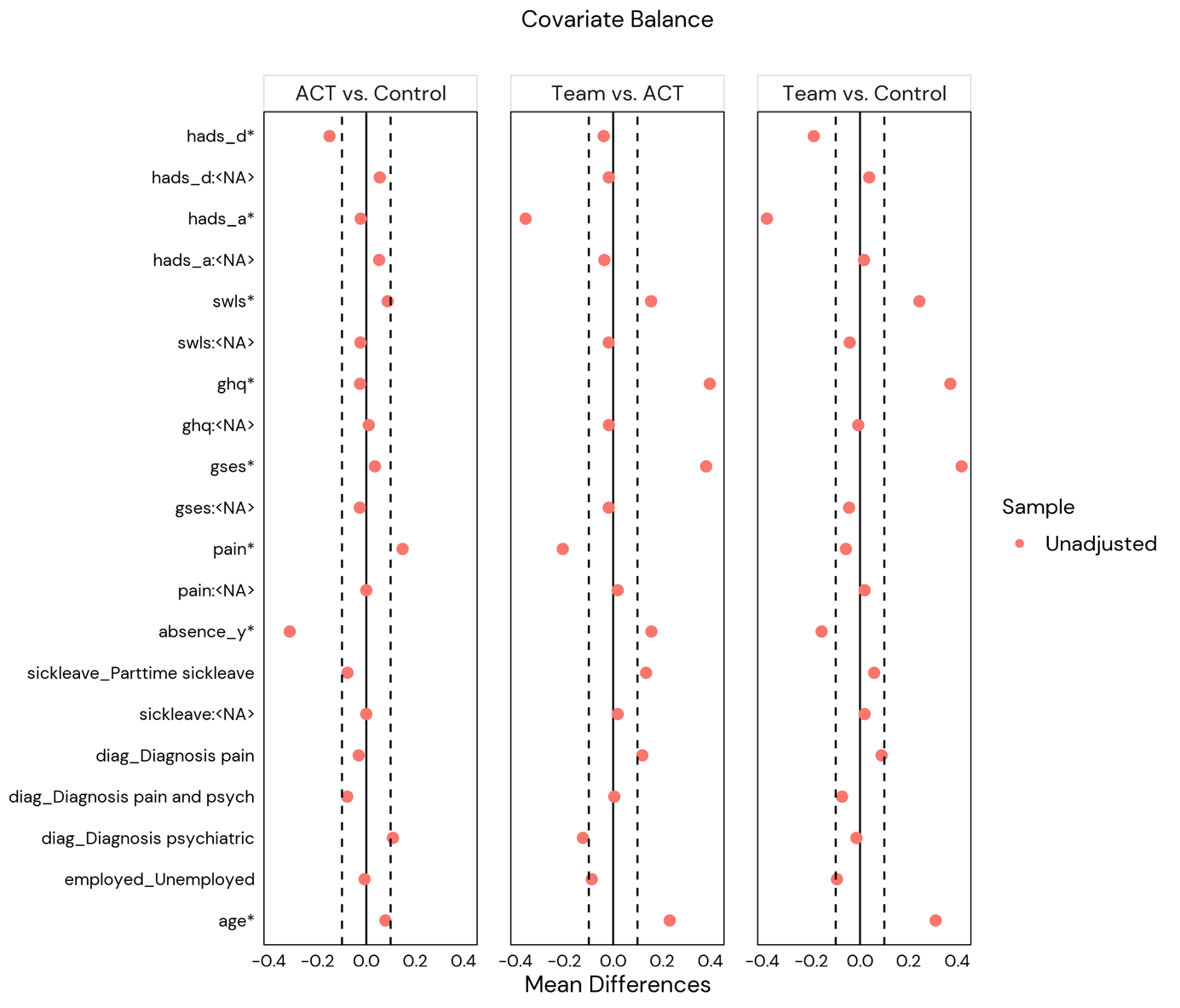


**Supplementary Figure 2.** Pre-treatment covariate balance plot for the participants in the 10-year follow-up for secondary outcomes.


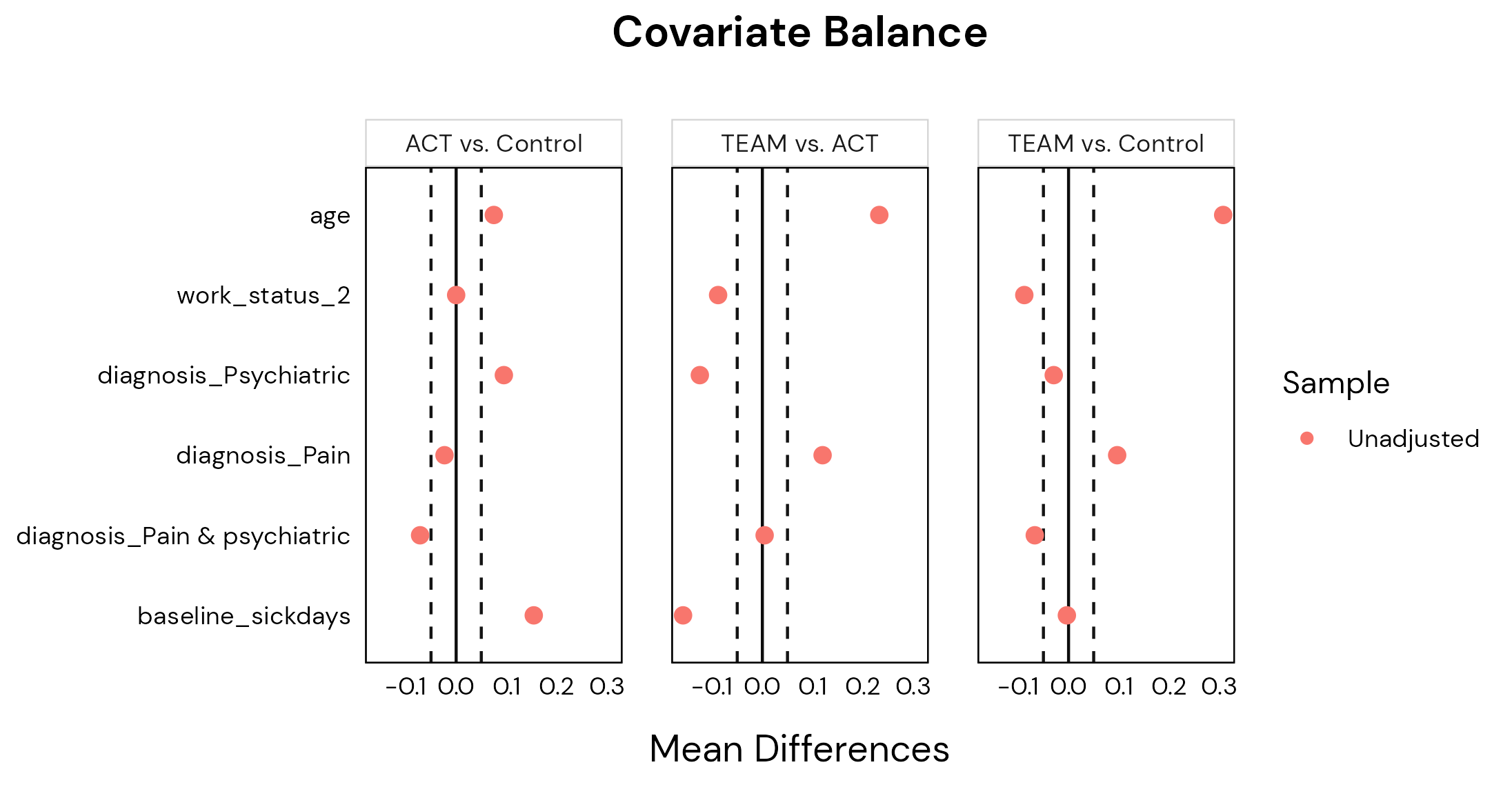


**Supplementary Figure 3.** Covariate balance plot at baseline for work disability days analysis.

### Drop-out analyses

Due to partial attrition, we conducted separate analyses for each of the six rating scale outcomes to investigate patterns of missingness at the 2- and 10-year follow-ups. For each outcome, a binary indicator variable was created to denote whether a response was missing. This variable served as the dependent variable in a series of robust Poisson regression models, with all baseline variables included as predictors. The aim was to assess whether any baseline characteristic was significantly associated with missingness, thereby suggesting that data may not be missing at random (NMAR). P-values were corrected for multiple comparisons using the Bonferroni-Holm method. No statistically significant predictors were identified. It is important to acknowledge that the absence of statistically significant predictors does not confirm that data are missing at random (MAR), as this analysis cannot definitively establish the missingness mechanism.

### Model specifications for primary and secondary outcomes

Example formula for cross-sectional ordered beta regression with the outcome work absence days during year 1:

glmmTMB(formula = y1_scaled ~ tx_group * ( age_c + employment_status + diagnosis),

data = d2,

family = “ordbeta”)

Below is an example MMRM formula for outcome GHQ. The only modifications between models are the outcome and the *outcome_pre_c* variable to add the grand mean centered pre-measurement of the outcome as a covariate interacting with the time variable.

mmrm(formula = ghq ~ ghq_pre_c*time + tx_group * (time + age_c + absence_years_c + sickleave + diagnosis + employment_status) + tx_group:ghq_pre_c + us(time|tx_group/id),
 data = d_mmrm,

method = "Kenward-Roger")

**Supplementary Table 2.** Estimated marginal mean contrasts for treatment groups’ cumulative work disability days each year until eight years follow-up.

| Year | Contrast | EMM and 95% CI [lower, upper] |
| --- | --- | --- |
| 1 | ACT - Control | -38.8 [-104.5, 34.9] |
|  | TEAM - Control | -32.8 [-97.4, 36.5] |
| 2 | ACT - Control | -63.8 [-169.5, 46.9] |
|  | TEAM - Control | -88.6 [-177.5, 12.2] |
| 3 | ACT - Control | -73.6 [-232.4, 87] |
|  | TEAM - Control | -133.3 [-276, 16.3] |
| 4 | ACT - Control | -110 [-323.9, 91.4] |
|  | TEAM - Control | -220.3 [-403.2, -21.9] |
| 5 | ACT - Control | -134.7 [-424.6, 117.9] |
|  | TEAM - Control | -218.8 [-440.6, 46.3] |
| 6 | ACT - Control | -216.4 [-552.1, 121.8] |
|  | TEAM - Control | -293.3 [-552.6, 10.1] |
| 7 | ACT - Control | -396.8 [-870.8, 91.5] |
|  | TEAM - Control | -398.6 [-774.3, -26.9] |
| 8 | ACT - Control | -450.1 [-907.7, 37.6] |
|  | TEAM - Control | -424.9 [-757.5, -4.6] |

| **Supplementary Table 3.** Differences in estimated marginal means regarding secondary outcome measures at one, two- and ten-years follow-up for ACT and TEAM compared with Control, respectively. | | | | | | | |
| --- | --- | --- | --- | --- | --- | --- | --- |
| Outcome | Time | Contrast | Estimate | SE | df | T-ratio | p-value |
| HADS-D | Year 1 | ACT - Control | -2.23 | 0.64 | 126.30 | -3.47 | 0.001* |
|  |  | Team - Control | -2.27 | 0.67 | 133.00 | -3.41 | 0.002* |
|  | Year 2 | ACT - Control | -2.12 | 0.72 | 109.20 | -2.95 | 0.008* |
|  |  | Team - Control | -0.95 | 0.69 | 118.20 | -1.38 | 0.291 |
|  | Year 10 | ACT - Control | -0.92 | 0.77 | 90.80 | -1.20 | 0.387 |
|  |  | Team - Control | -1.10 | 0.79 | 101.90 | -1.39 | 0.290 |
| HADS-A | Year 1 | ACT - Control | -1.88 | 0.72 | 122.40 | -2.62 | 0.019* |
|  |  | Team - Control | -2.21 | 0.67 | 138.20 | -3.28 | 0.003* |
|  | Year 2 | ACT - Control | -2.76 | 0.80 | 116.10 | -3.46 | 0.001* |
|  |  | Team - Control | -2.08 | 0.72 | 119.50 | -2.89 | 0.009* |
|  | Year 10 | ACT - Control | -0.23 | 0.91 | 94.60 | -0.25 | 0.942 |
|  |  | Team - Control | -0.85 | 0.86 | 97.70 | -0.99 | 0.511 |
| SWLS | Year 1 | ACT - Control | 3.04 | 1.06 | 123.10 | 2.88 | 0.009* |
|  |  | Team - Control | 3.62 | 0.97 | 138.80 | 3.71 | 0.001* |
|  | Year 2 | ACT - Control | 1.95 | 1.18 | 99.10 | 1.66 | 0.181 |
|  |  | Team - Control | 1.45 | 0.94 | 121.50 | 1.54 | 0.222 |
|  | Year 10 | ACT - Control | 1.18 | 1.29 | 98.40 | 0.92 | 0.560 |
|  |  | Team - Control | 1.58 | 1.20 | 105.70 | 1.32 | 0.325 |
| GHQ | Year 1 | ACT - Control | 2.87 | 1.18 | 128.10 | 2.43 | 0.032* |
|  |  | Team - Control | 4.37 | 1.13 | 144.40 | 3.88 | <0.001* |
|  | Year 2 | ACT - Control | 1.85 | 0.96 | 112.60 | 1.92 | 0.107 |
|  |  | Team - Control | 1.51 | 0.88 | 105.40 | 1.71 | 0.163 |
|  | Year 10 | ACT - Control | 0.14 | 1.27 | 86.30 | 0.11 | 0.985 |
|  |  | Team - Control | 2.16 | 1.27 | 98.40 | 1.70 | 0.166 |
| GSES | Year 1 | ACT - Control | 1.16 | 1.16 | 123.90 | 1.00 | 0.504 |
|  |  | Team - Control | 1.95 | 1.10 | 121.30 | 1.77 | 0.143 |
|  | Year 2 | ACT - Control | 1.20 | 1.25 | 96.60 | 0.96 | 0.532 |
|  |  | Team - Control | 1.83 | 1.15 | 92.90 | 1.59 | 0.206 |
|  | Year 10 | ACT - Control | -0.07 | 1.22 | 93.20 | -0.06 | 0.995 |
|  |  | Team - Control | 2.02 | 1.24 | 95.60 | 1.63 | 0.191 |
| PAIN | Year 1 | ACT - Control | -0.74 | 0.41 | 110.60 | -1.82 | 0.131 |
|  |  | Team - Control | -0.14 | 0.39 | 134.50 | -0.36 | 0.896 |
|  | Year 2 | ACT - Control | -0.55 | 0.39 | 63.50 | -1.41 | 0.281 |
|  |  | Team - Control | -0.32 | 0.33 | 91.10 | -0.97 | 0.523 |
|  | Year 10 | ACT - Control | 1.04 | 0.50 | 79.60 | 2.09 | 0.074 |
|  |  | Team - Control | -0.12 | 0.48 | 97.50 | -0.25 | 0.944 |
| Note: SE = Standard Error; HADS-D = Hospital Anxiety Depression Scale - Depression; HADS-A = Hospital Anxiety Depression Scale - Anxiety; SWLS = Satisfaction With Life Scale; GHQ = General Health Questionnaire; GSES = General Self-Efficacy Scale; PAIN = Örebro Musculoskeletal Pain Scale; * = p-value < .05; For contrasts, *p*-value adjustments were made using Dunnett’s method for two tests. | | | | | | | |


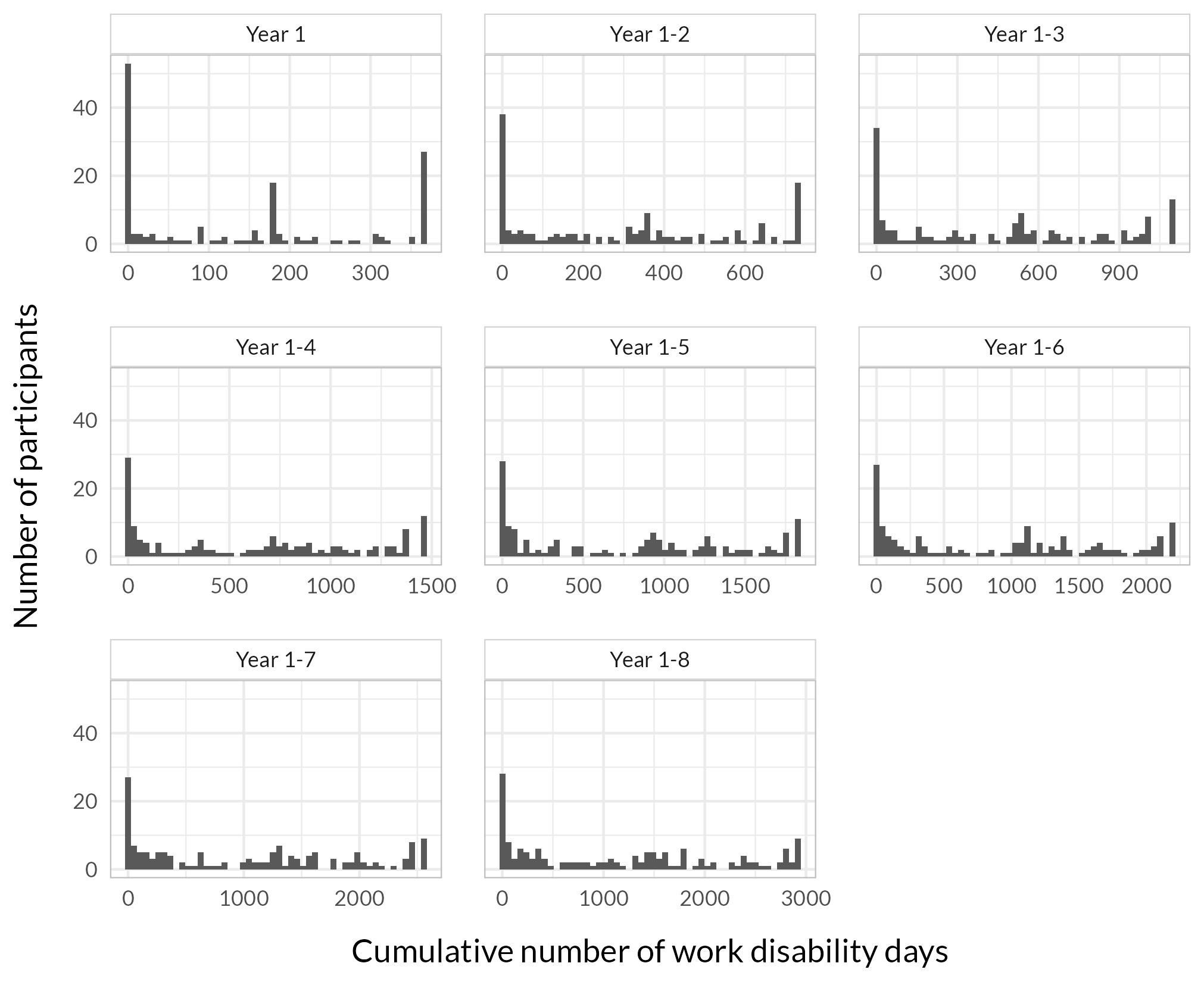


**Supplementary Figure 4.** Observed data distributions showing cumulative number of work disability days over time.


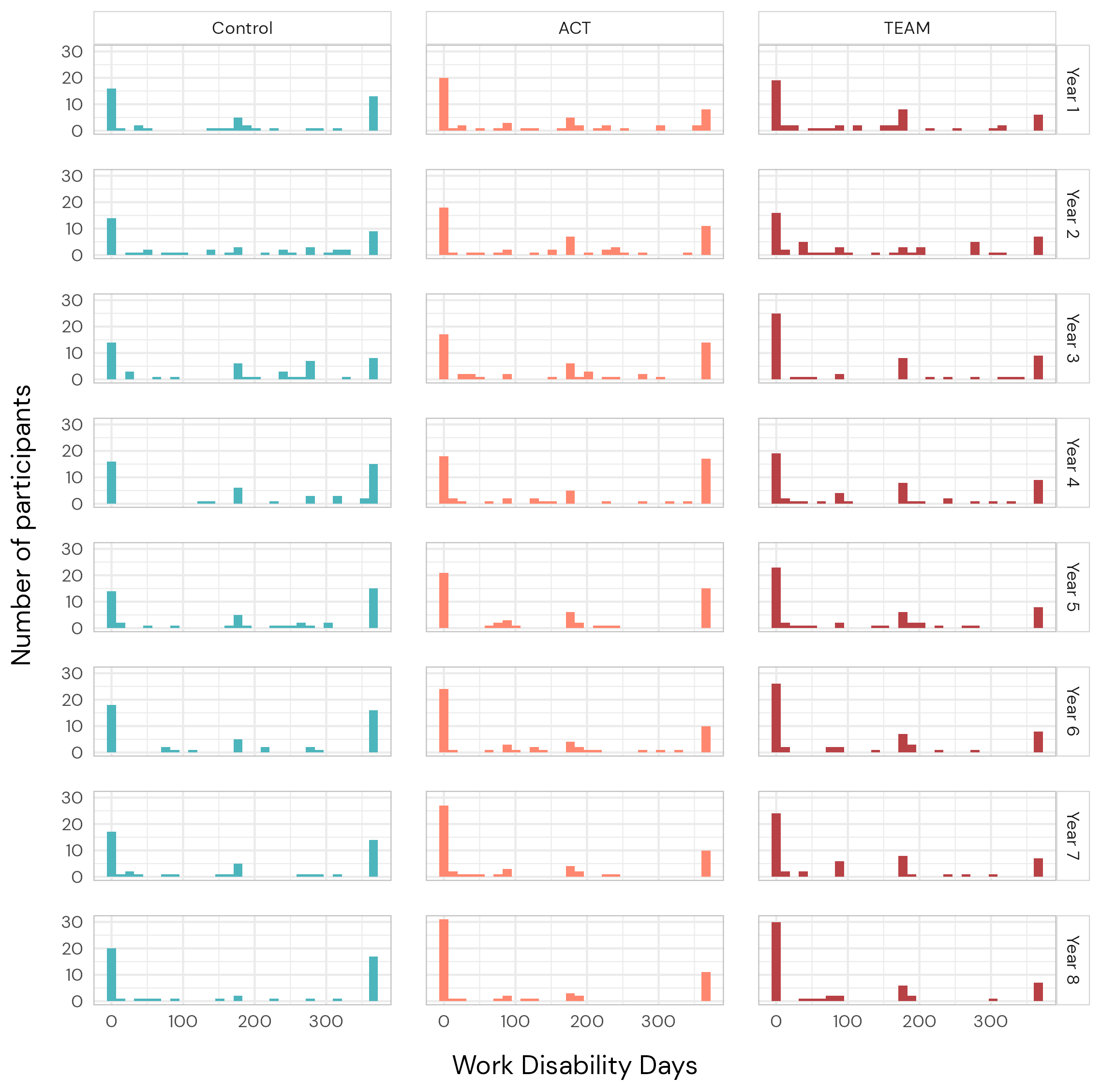


**Supplementary Figure 5.** Observed data distributions showing number of work disability days per year by treatment group.

### Sensitivity analysis for secondary outcomes

Since our MMRM models estimate many coefficients with a relatively limited sample size, we also conducted a sensitivity analysis using overlap weights in cross-sectional (yearly) models. We chose overlap weights (OW) based on comparing the effective sample size to inverse probability of treatment weighting (IPTW) and the superior stability and performance of OW (Zeng, et al., 2021). Separate OW regression models were used for each outcome and year, using the same set of covariates as in the MMRM analysis, but without interactions since the OW regression uses the treatment group as outcome. Weights were estimated using the R package `WeightIt` (Greifer, 2025). The outcome regression was fit using the function `lm_weightit()`, which uses M-estimation for standard errors, only using treatment group interacted with the pre-measurement (using a natural spline with three degrees of freedom) as predictor variables. Results are reported in Supplementary Table 4 and Supplementary Figure 6. Example model specification for one outcome at year 1:

w_t1_ato <- WeightIt::weightit(tx_group ~ absence_years_c + sickleave + diagnosis + employment_status + age_c + outcome_pre_c,

method = "glm", link = "logit", estimand = "ATO"

)

t1_outcome <- WeightIt::lm_weightit(outcome ~ tx_group * splines::ns(outcome_pre, df = 3),

vcov = "asympt", x = T, y = T,

weightit = w_t1_ato)

avg_comparisons(t1_outcome, variables = "tx_group", wts = w_t1_ato$weights)

**Supplementary Table 4.** Estimated group contrasts from cross-sectional models using overlap weights for secondary outcome measures at one, two- and ten-years follow-up for ACT and TEAM compared with Control, respectively.

| Outcome | Time | Contrast | Estimate | SE | p-value |
| --- | --- | --- | --- | --- | --- |
| HADS-D | Year 1 | ACT - Control | -1.94 | 0.61 | 0.001* |
|  |  | Team - Control | -2.55 | 0.60 | <0.001* |
|  | Year 2 | ACT - Control | -1.61 | 0.65 | 0.013* |
|  |  | Team - Control | -0.68 | 0.69 | 0.323 |
|  | Year 10 | ACT - Control | -0.47 | 0.67 | 0.479 |
|  |  | Team - Control | -0.58 | 0.73 | 0.421 |
| HADS-A | Year 1 | ACT - Control | -1.14 | 0.62 | 0.067 |
|  |  | Team - Control | -2.50 | 0.60 | <0.001* |
|  | Year 2 | ACT - Control | -1.83 | 0.72 | 0.011* |
|  |  | Team - Control | -1.92 | 0.65 | 0.003* |
|  | Year 10 | ACT - Control | 0.25 | 0.73 | 0.735 |
|  |  | Team - Control | -0.91 | 0.69 | 0.186 |
| SWLS | Year 1 | ACT - Control | 2.01 | 0.96 | 0.036* |
|  |  | Team - Control | 3.36 | 0.84 | <0.001* |
|  | Year 2 | ACT - Control | 1.34 | 1.04 | 0.198 |
|  |  | Team - Control | 0.69 | 0.81 | 0.396 |
|  | Year 10 | ACT - Control | -0.04 | 1.15 | 0.974 |
|  |  | Team - Control | 1.59 | 1.12 | 0.157 |
| GHQ | Year 1 | ACT - Control | 2.49 | 1.07 | 0.021* |
|  |  | Team - Control | 3.99 | 1.09 | <0.001* |
|  | Year 2 | ACT - Control | 1.58 | 0.94 | 0.093 |
|  |  | Team - Control | 1.71 | 0.85 | 0.046* |
|  | Year 10 | ACT - Control | -0.53 | 1.04 | 0.615 |
|  |  | Team - Control | 1.40 | 1.13 | 0.214 |
| GSES | Year 1 | ACT - Control | 0.65 | 1.03 | 0.531 |
|  |  | Team - Control | 2.29 | 0.97 | 0.019* |
|  | Year 2 | ACT - Control | 1.50 | 1.18 | 0.201 |
|  |  | Team - Control | 1.82 | 1.18 | 0.123 |
|  | Year 10 | ACT - Control | -0.53 | 1.09 | 0.625 |
|  |  | Team - Control | 1.72 | 1.12 | 0.127 |
| PAIN | Year 1 | ACT - Control | -0.61 | 0.37 | 0.097 |
|  |  | Team - Control | -0.35 | 0.39 | 0.357 |
|  | Year 2 | ACT - Control | -0.29 | 0.31 | 0.354 |
|  |  | Team - Control | -0.56 | 0.31 | 0.075 |
|  | Year 10 | ACT - Control | 0.97 | 0.47 | 0.038* |
|  |  | Team - Control | 0.29 | 0.45 | 0.528 |
| Note: SE = Standard Error; HADS-D = Hospital Anxiety Depression Scale - Depression; HADS-A = Hospital Anxiety Depression Scale - Anxiety; SWLS = Satisfaction With Life Scale; GHQ = General Health Questionnaire; GSES = General Self-Efficacy Scale; PAIN = Örebro Musculoskeletal Pain Scale; * = p-value < .05 | | | | | |

**Supplementary Figure 6.** Estimated group contrasts from cross-sectional models using overlap weights for secondary outcome measures at one, two- and ten-years follow-up for ACT and TEAM compared with Control, respectively.


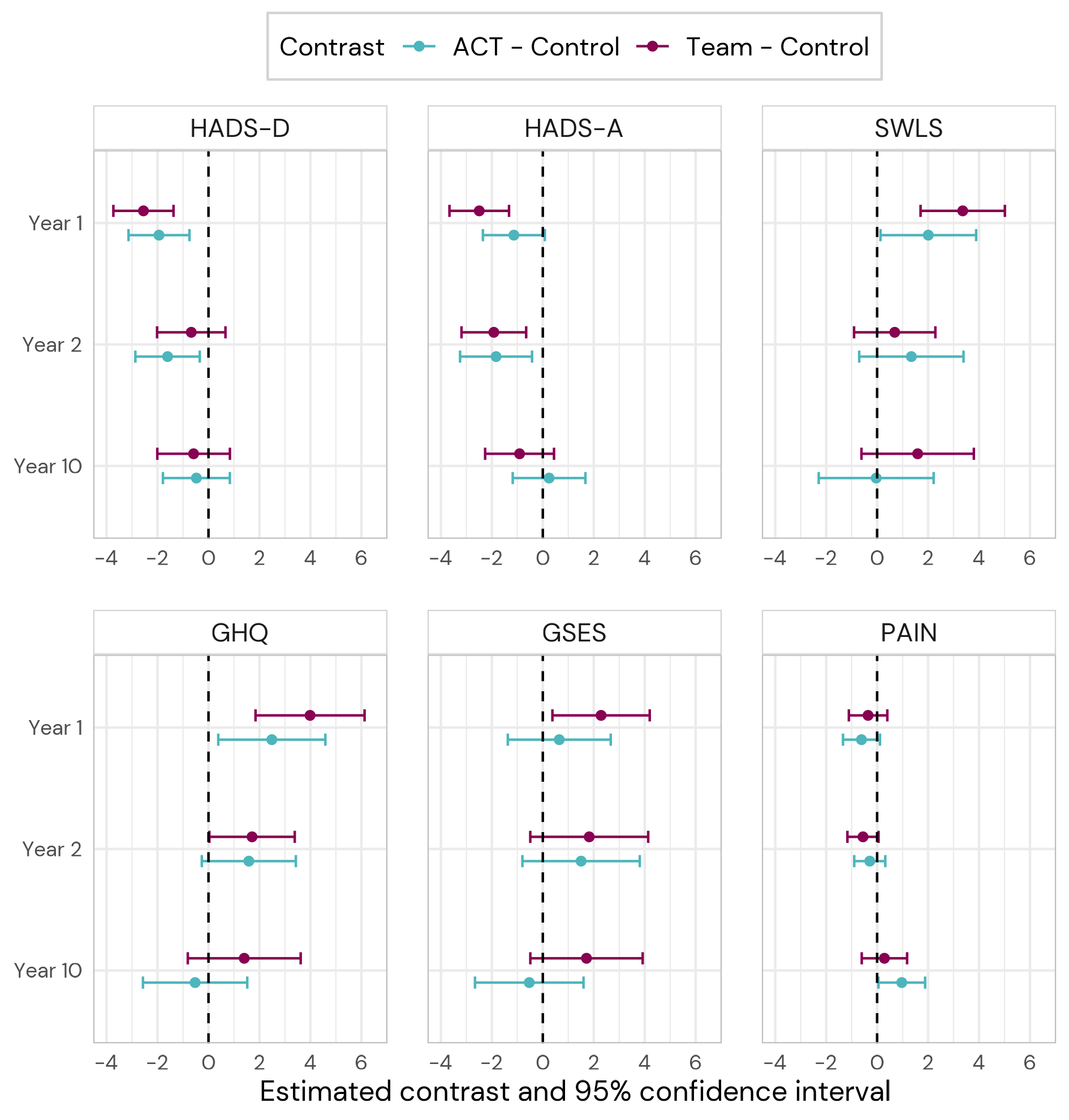


# References

Greifer, N. (2026). cobalt: Covariate Balance Tables and Plots [Computer software]. <https://doi.org/10.32614/CRAN.package.cobalt>

Greifer, N. (2025). WeightIt: Weighting for Covariate Balance in Observational Studies [Computer software]. <https://doi.org/10.32614/CRAN.package.WeightIt>

Zeng, S., Li, F., Wang, R., & Li, F. (2021). Propensity score weighting for covariate adjustment in randomized clinical trials. Statistics in Medicine, 40(4), 842–858. <https://doi.org/10.1002/sim.8805>
